# Sex-Dependent Brain Plasticity in Neurological Disease: From Biological Variability to Adaptive, Compensatory, and Maladaptive Trajectories

**DOI:** 10.3390/biology15141176

**Published:** 2026-07-17

**Authors:** Alessandro Avitabile, Dario Rusciano, Roberta Amato, Ludovica Cannizzaro, Caterina Gagliano

**Affiliations:** 1 Neurovisual Science Technology, Research Division (NEST-G.R.I.D.O.), 95125 Catania, Italy; alessandro.avitabile@unipa.it (A.A.); robertaamato@nestweb.it (R.A.); ludocann@gmail.com (L.C.); 2 Biomedicine, Neuroscience and Advance Diagnostics (BIND) Department, University of Palermo, 90128 Palermo, Italy; 3 Faculty of Medicine and Surgery, Kore University, 94100 Enna, Italy; caterina.gagliano@unikore.it; 4 Morgagni Ophthalmology Clinic, 95125 Catania, Italy

**Keywords:** brain plasticity, biological sex, sex differences, sex and gender, adaptive plasticity, compensatory plasticity, maladaptive plasticity, adaptive reserve, neuroinflammation, mitochondrial function, epigenetic regulation, neurodevelopment, neurodegeneration, rehabilitation

## Abstract

Brain plasticity is often described as the capacity of the nervous system to change in response to development, experience, injury, disease, or treatment. That definition is useful, but it can obscure two clinically important points: plasticity is biologically constrained, and change is not always beneficial. This narrative review examines sex-dependent brain plasticity as a context-sensitive process rather than as a simple male–female contrast. We distinguish four operational outcomes of plasticity: (i) reparative plasticity, which restores structure or function; (ii) compensatory plasticity, which preserves performance through alternative or more costly strategies; (iii) insufficient plasticity, in which reorganization is too weak or unstable to sustain function; and (iv) maladaptive plasticity, in which plastic change reinforces dysfunction, pain, excitability, rigidity, or decline. We also define adaptive reserve as the integrated capacity of neural, glial, vascular, immune, metabolic, endocrine, and gene-regulatory systems to support useful reorganization under stress. The review evaluates endocrine, synaptic, neuroimmune, mitochondrial, vascular, stress-related, and epigenetic mechanisms, indicating where evidence for sex-dependent effects is relatively strong and where it remains indirect, inconsistent, or context-dependent. Disease examples include autism spectrum disorder, attention-deficit/hyperactivity disorder, epilepsy, intellectual disability, Alzheimer’s disease, Parkinson’s disease, amyotrophic lateral sclerosis, Huntington’s disease, traumatic and ischemic injury, multiple sclerosis, chronic pain, aging, and systemic metabolic or inflammatory disorders. Throughout, biological sex is separated from gender-related social, diagnostic, and health-care determinants. We conclude that therapeutic strategies should not aim simply to enhance plasticity, but to guide it by matching intervention, timing, dose, biological readiness, and monitoring to the patient’s adaptive state.

## Simple Summary

The brain can change during development, aging, disease, and recovery, but this capacity is not always helpful in the same way. Some plastic responses restore function, some preserve performance through compensation, some remain insufficient, and others reinforce harmful patterns such as pain, abnormal excitability, or inefficient strategies. This review examines how biological sex may influence these outcomes without reducing the brain to fixed male and female categories. Sex-related biology can affect hormones, immune responses, metabolism, vascular support, stress regulation, gene activity, and the clinical conditions in which plasticity takes place. In addition, the review also distinguishes biological sex from gender-related factors such as diagnostic delay, care access, and social expectations. A more precise understanding of sex-dependent plasticity may help explain why similar diseases or treatments produce different outcomes and may support more realistic therapeutic strategies for guiding brain recovery.

## 1. Introduction: Brain Plasticity as a Sex-Dependent Biological Process

The inclusion of sex as a biological variable has become a central requirement of modern biomedical research, especially in preclinical neuroscience, where single-sex designs can conceal mechanisms that later affect translation [1,2,3]. At the same time, the Sex and Gender Equity in Research (SAGER) recommendations have emphasized that sex and gender should be reported and interpreted explicitly, not used interchangeably [4]. This point is particularly important in plasticity research because experimental animals, cellular models, and human patients do not carry the same mixture of chromosomal, hormonal, social, behavioral, and health-care influences.

In this review, “sex” refers primarily to biological variables such as sex chromosomes, gonadal organization, hormonal milieu, reproductive stage, and sex-linked physiological regulation. “Gender” refers to social roles, identity, behavior, expectations, exposure, diagnostic pathways, and access to care. The two domains interact in human disease, but they are not the same. We therefore use “sex-dependent” when discussing biological mechanisms and “sex/gender-related” when clinical observations may reflect a mixture of biological and sociocultural determinants [4,5,6,7].

Brain plasticity is usually described as the ability of the nervous system to change. That definition is correct, but incomplete. A plastic response can support development, learning, repair, and rehabilitation; it can also stabilize inefficient compensation, amplify pain, sustain abnormal excitability, or fail when the tissue environment is no longer permissive [8,9,10]. The relevant question is therefore not only whether plasticity is increased or decreased, but whether it is directed toward a useful and sustainable functional state.

Sex can shape this process without dividing the brain into two rigid categories. Sex-related biology may influence synaptic excitability, dendritic remodeling, neurotrophic signaling, microglial and astrocytic responses, mitochondrial function, vascular support, epigenetic regulation, and stress-axis reactivity [5,11,12,13,14,15,16,17,18,19,20,21,22,23]. These mechanisms do not operate as isolated modules. A synaptic change has different consequences if it occurs in a tissue with controlled inflammation, adequate energy supply, and stable vascular support than if it occurs during chronic stress, metabolic insufficiency, or progressive neurodegeneration.

The framework therefore treats sex-dependent plasticity as a trajectory. Plasticity has thresholds, because a stimulus must reach a permissive biological state before change begins. It has direction, because the circuits recruited may support recovery or reinforce dysfunction. It has cost, because reorganization requires energy, vascular support, sleep, and immune regulation. It has persistence, because transient changes must be stabilized to become clinically meaningful. Finally, it can fail, producing insufficient or maladaptive outcomes in brain injury, neurodegeneration, chronic pain, and other pathological states [23,24,25,26,27]. Stress regulation also belongs to this architecture because hypothalamic–pituitary–adrenal (HPA) axis responses differ by sex and can influence plasticity, inflammation, and metabolic load [28].

The aim of this review is to move beyond a purely theoretical discussion. We define classification criteria, identify where evidence is strong or weak, include opposing and neutral findings, discuss detailed disease examples, and translate the expression “guiding plasticity” into practical implications for rehabilitation, neuromodulation, pharmacological treatment, and study design.

### 1.1. Literature Search and Review Strategy

This is a narrative, mechanistically oriented review rather than a systematic review or meta-analysis. The literature was searched in PubMed, Scopus, and Google Scholar. The main search period covered publications from January 2010 to February 2026, with earlier foundational papers retained when they established concepts that remain central to neuroplasticity, maladaptive plasticity, rehabilitation, or sex-as-a-biological-variable methodology. Searches were performed using combinations of: “brain plasticity”, “neuroplasticity”, “sex differences”, “biological sex”, “sex as a biological variable”, “gender”, “synaptic plasticity”, “BDNF”, “microglia”, “neuroinflammation”, “mitochondria”, “vascular function”, “epigenetics”, “stress axis”, “adaptive reserve”, “rehabilitation”, “stroke”, “traumatic brain injury”, “Alzheimer’s disease”, “Parkinson’s disease”, “amyotrophic lateral sclerosis”, “Huntington’s disease”, “autism spectrum disorder”, “ADHD”, “epilepsy”, “intellectual disability”, “multiple sclerosis”, “chronic pain”, and “aging”.

Articles were included when they met at least one of four criteria: (i) they directly examined sex-dependent biological mechanisms relevant to brain plasticity; (ii) they provided clinical or preclinical evidence of plasticity-related adaptation, compensation, failure, or maladaptation; (iii) they addressed sex/gender reporting or study-design standards; or (iv) they provided mechanistic context necessary to interpret disease-related plasticity. Reviews were used to frame broad areas, while original experimental or clinical studies were prioritized when they provided more specific evidence. Studies were excluded from detailed discussion when sex was only mentioned descriptively, when the outcome was unrelated to plasticity or adaptive reorganization, or when methodological details were insufficient to interpret sex/gender effects.

Because the review is narrative, no formal risk-of-bias scoring or pooled quantitative analysis was performed. Instead, evidence was appraised qualitatively and grouped as relatively strong, moderate, indirect, or inconsistent, depending on the degree to which sex-dependent effects were supported across models, brain regions, disease stages, and outcome levels. This explicit evidence appraisal is important to avoid presenting conceptual links as proven causal pathways.

### 1.2. Operational Definitions: Plasticity Classes and Adaptive Reserve

A central point concerns terminology. Adaptive, compensatory, insufficient, and maladaptive plasticity should not be used as broad labels alone. These terms can be defined by criteria that are applicable to experimental and clinical data. The classification is not intended to create rigid categories; the same process may change meaning across disease stage, time, and outcome level. Still, operational criteria are useful because they force the reader to ask what function a plastic response serves, how durable it is, and whether it improves or worsens the patient’s adaptive state.

Adaptive reserve is defined here as the integrated capacity of neural, glial, vascular, immune, metabolic, endocrine, and gene-regulatory systems to support useful reorganization under biological stress. It differs from brain reserve, which usually refers to structural capacity such as brain size, synapse number, or network redundancy; from cognitive reserve, which refers to the ability to maintain performance through efficient or flexible cognitive strategies; and from resilience, which is a broader outcome concept describing preserved function despite adversity. Adaptive reserve is more mechanistic and dynamic: it concerns whether the organism is able to initiate, support, and stabilize plastic change when challenged (Table 1).

## 2. Biological Architecture of Sex-Dependent Brain Plasticity

**Figure 1:**
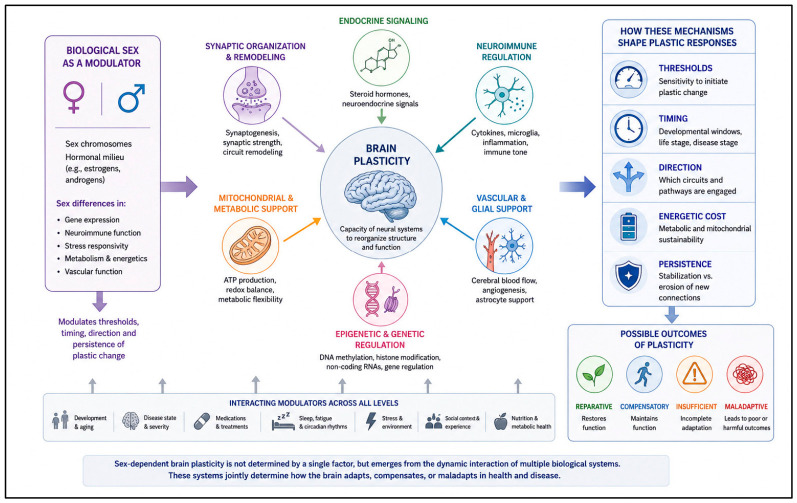
Biological architecture of sex-dependent brain plasticity. The figure is a conceptual synthesis. Solid arrows represent relationships supported by the cited literature, whereas the overall arrangement should not be read as proof of direct causality among all components. Biological sex can modulate endocrine signaling, synaptic remodeling, neuroimmune regulation, mitochondrial and metabolic support, vascular and glial function, stress biology, and epigenetic regulation. These mechanisms interact with age, disease state, sleep, stress, medication exposure, environment, and metabolic health. Together, they influence plasticity thresholds, timing, direction, energetic cost, and persistence, resulting in reparative, compensatory, insufficient, or maladaptive outcomes.

The biological architecture of sex-dependent plasticity is best understood as a set of interacting layers. It is not sufficient to list endocrine, synaptic, immune, metabolic, vascular, stress-related, and epigenetic mechanisms as parallel influences. Their relevance depends on the strength of evidence, the brain region considered, disease stage, age, hormonal condition, and the outcome being measured. For this reason, the following sections separate relatively well-supported mechanisms from more indirect or context-dependent ones and indicate where the literature remains mixed (Table 2 and Figure 1).

### 2.1. Endocrine and Synaptic Mechanisms: Relatively Strong but Region-Dependent Evidence

Sex steroids are among the most direct biological modulators of plasticity. Estrogens, progesterone, and androgens can affect neuronal excitability, dendritic architecture, glutamatergic transmission, receptor trafficking, neurogenesis, and neurotrophin signaling [5,11,12]. These effects are not uniform across the brain. The same hormone may have different consequences in hippocampus, cortex, striatum, hypothalamus, or spinal cord, and the direction of the effect may depend on developmental stage, receptor distribution, disease state, and timing of exposure.

BDNF is a useful example. It is often treated as a generic marker of plasticity, but its biological meaning changes with region, receptor balance, endocrine state, stress exposure, inflammation, metabolic condition, and age [12]. A rise in BDNF may support learning or repair in one setting and reflect compensatory stress in another. For this reason, BDNF and related neurotrophic signals are informative only when interpreted with timing, tissue, and function.

### 2.2. Neuroimmune Mechanisms: Strong Biological Rationale, Variable Direction

Microglia, astrocytes, cytokines, complement signaling, and inflammasome-related pathways influence synaptic pruning, neurogenesis, tissue repair, and network remodeling. Sex differences in immune maturation and microglial phenotype have been described across development, adulthood, aging, and disease [14,15,16,17]. This provides a strong biological rationale for sex-dependent plasticity, particularly when plasticity unfolds under inflammatory pressure.

The evidence is nevertheless not one-directional. It is not accurate to say that one sex is simply more inflammatory or more plastic. Microglial responses differ by brain region, developmental stage, gonadal hormone history, early-life immune experience, and disease model [14,16,17]. Controlled immune activity may support repair, whereas persistent inflammation may restrict plasticity or convert adaptive responses into maladaptive ones. This is the main reason why the figures and the text avoid implying linear causality.

### 2.3. Mitochondrial, Metabolic, and Vascular Support: Strong Relevance, Often Indirect Sex-Specific Evidence

Plasticity is energetically expensive. Synaptic remodeling, axonal sprouting, receptor trafficking, remyelination, glial regulation, angiogenesis, and gene expression require mitochondrial function and vascular support. Mitochondrial dysfunction is strongly implicated in brain aging and neurodegenerative disease [18,19]. Sex-specific evidence is more uneven, but experimental work in Alzheimer’s disease models shows that sex and age can shape hypothalamic bioenergetics and inflammatory signaling [20].

The implication is important but should be stated carefully. Metabolic and mitochondrial factors do not prove sex-dependent plasticity by themselves. They define the biological resources that make plasticity sustainable. If energetic reserve is reduced, a plastic response may be initiated but not maintained. If vascular supply is unstable, a rehabilitation-induced or stimulation-induced change may fail to consolidate. Sex may influence these constraints, but disease stage and systemic health may be equally important.

### 2.4. Epigenetic and Gene-Regulatory Mechanisms: Plausible Mechanisms for Persistence

For plasticity to matter clinically, transient molecular changes must often become durable. Epigenetic regulation, including chromatin remodeling, DNA methylation-related processes, histone modifications, and non-coding RNAs, provides one route through which development, stress, inflammation, injury, and treatment can leave persistent biological traces [21,22]. Sex chromosomes, gonadal hormones, early-life endocrine exposure, immune signaling, and metabolism can all interact with these regulatory systems [13,14].

In neurodegenerative disease, sex-related epigenetic mechanisms have been proposed as contributors to vulnerability, compensation, and progression, although the evidence is still more developed for disease association than for direct demonstration of plasticity mechanisms [22]. Thus, epigenetic regulation should be considered a plausible route for the persistence of sex-dependent plasticity, not a universal explanation for it.

### 2.5. Stress Biology, Context Dependence, and Neutral Evidence

Stress regulation is treated as a separate mechanism rather than as a background variable. Sex differences in HPA-axis responses and the influence of gonadal hormones on stress biology are well documented [28]. Stress can change plasticity through glucocorticoid signaling, sleep disruption, inflammatory tone, mitochondrial demand, and behavioral avoidance. In rehabilitation and chronic pain, these factors may determine whether stimulation or practice consolidates into useful change or remains transient.

Equally important, not all studies find sex differences at the final behavioral endpoint. A neutral result may mean that sex is not relevant in that model, but it may also mean that the endpoint was insensitive to pathway differences, that males and females reached the same outcome through different routes, or that the timing of measurement missed divergent trajectories. Interpretation should therefore avoid two errors: ignoring sex altogether and exaggerating every sex-related difference into a deterministic rule.

## 3. Sex-Dependent Plasticity Across Pathological Contexts

Pathology is not merely the background against which plasticity occurs. Disease changes the conditions under which plasticity develops. It can alter the tissue environment, available energy, immune tone, vascular supply, stress physiology, sleep, motivation, and behavioral opportunity. The following sections use specific disease examples to show how sex-dependent biology may shape plasticity under different forms of constraint.

### 3.1. Neurodevelopmental Disorders: Plasticity While Circuits Are Being Built

Autism spectrum disorder (ASD) and attention-deficit/hyperactivity disorder (ADHD) are discussed because they illustrate plasticity during circuit formation rather than plasticity after a completed brain has been injured. ASD involves developmental differences in social communication, sensory processing, behavioral flexibility, and, in some individuals, camouflaging or masking strategies that may delay diagnosis, especially in females [29,30,31]. ADHD involves the maturation of attentional, executive, reward, and emotional-regulation networks, with sex and gender influencing symptom recognition, comorbidity, and impairment across the lifespan [32,33].

ASD and ADHD do not exhaust the neurodevelopmental field. A wider review of sex and gender in neurodevelopmental conditions emphasizes that prevalence, clinical presentation, diagnosis, and support needs vary across disorders and may be shaped by both biological and social determinants [33].

Epilepsy is also relevant because sex hormones can influence neuronal excitability, seizure threshold, and responses to antiseizure medications [34,35]. Catamenial seizure patterns show that endocrine state can modulate network excitability in some patients, although not all epilepsy syndromes show the same degree of hormonal sensitivity [34].

Intellectual disability again illustrates the limits of simple sex comparisons. Some causes are linked to sex chromosomes, but many are not. In these conditions, sex may influence developmental timing, comorbid epilepsy, behavior, care access, and adaptive functioning, but direct evidence linking sex to plasticity mechanisms is still limited [36].

These examples show why neurodevelopmental plasticity should be considered as a trajectory. Compensation may begin early, before diagnosis, and may later become costly or insufficient when developmental demands increase. In human studies, however, it is difficult to separate biological sex from gendered expectations, differential referral, access to support, and social masking. Clinical sex ratios therefore cannot be interpreted as direct proof of biological plasticity.

### 3.2. Neurodegenerative Disease: Plasticity Under Progressive Constraint

In Alzheimer’s disease, synaptic dysfunction and compensatory plasticity can preserve function for a time, but compensation becomes progressively more costly as pathology, inflammation, vascular dysfunction, and mitochondrial stress accumulate [25]. Sex/gender differences in Alzheimer’s disease have been discussed in relation to genetics, hormonal history, inflammation, metabolism, and social determinants of health [37]. The central argument is not that one sex has uniformly more or less plasticity. Rather, sex-related biology may influence how compensation is built, when it fails, and which biological supports become limiting.

Parkinson’s disease provides a different model. Dopaminergic loss reshapes cortico-striatal learning, motor adaptation, reward processing, sleep, mood, autonomic function, and pain. Sex differences in Parkinson’s disease have been reported in epidemiology, phenotype, non-motor burden, and treatment-related features [38]. These observations are relevant to plasticity because the same dopaminergic deficit may be buffered by different endocrine, metabolic, and circuit contexts.

Amyotrophic lateral sclerosis (ALS) and Huntington’s disease (HD) extend further this discussion. ALS shows sex-related differences in incidence, phenotype, and biology, including interactions among motor neurons, skeletal muscle, metabolism, inflammation, and endocrine context [39]. In HD, sex-related clinical differences have been observed in large datasets, although evidence remains heterogeneous and should not be overgeneralized [40]. Both disorders illustrate an important limit of the plasticity concept: progressive degeneration can initially recruit compensation, but adaptive reserve may become exhausted as motor, metabolic, and cognitive burden increase.

### 3.3. Traumatic and Ischemic Injury: Repair, Compensation, and Maladaptation

Traumatic brain injury (TBI) and stroke make the distinction among reparative, compensatory, insufficient, and maladaptive plasticity clinically visible. TBI can involve axonal injury, focal lesions, neuroinflammation, vascular dysfunction, endocrine disruption, sleep disturbance, fatigue, pain, and affective symptoms. Sex differences after TBI have been reported, but their direction varies with injury severity, age, model, outcome, and clinical context [41]. This variability argues for trajectory-based interpretation rather than broad claims that one sex recovers better.

Stroke recovery depends on spontaneous biological repair, peri-lesional reorganization, motor learning, neurovascular adaptation, and rehabilitation-driven practice [26]. Sex differences in functional outcome after stroke are influenced by age, comorbidities, severity, access to care, and biological factors [42]. Here, sex/gender separation is crucial: worse outcomes in older women, for example, may reflect biological variables, social support, pre-stroke disability, access to rehabilitation, or combinations of these factors.

Maladaptive plasticity after injury includes abnormal excitability, seizures, spasticity, chronic pain, learned non-use, and inefficient network recruitment. Conversely, compensatory plasticity can preserve function but become too costly if it prevents more efficient recovery. For this reason, the therapeutic task is not simply to stimulate plasticity, but to shape timing, intensity, fatigue management, pain control, and consolidation.

### 3.4. Aging, Multiple Sclerosis, Chronic Pain, and Systemic Disease

Aging does not abolish plasticity. It changes its biological economy. Older brains can learn and compensate, but plastic responses may become slower, more vulnerable to inflammation, more dependent on vascular support, and more energetically costly. Sex-related differences in endocrine aging, immune aging, mitochondrial function, and vascular regulation can influence the point at which compensation becomes insufficient [43].

Multiple sclerosis (MS) combines inflammation, demyelination, neurodegeneration, fatigue, and variable recovery. Recent work suggests that the relationship between MRI disease burden and neurofunctional performance may differ by sex, indicating that damage and function are not always linked in the same way [44]. Chronic pain is another example in which plasticity can become maladaptive: circuits may remain sensitized after tissue healing, and neuromodulation or behavioral therapy must redirect rather than simply amplify plasticity [27].

Systemic disease also matters. Diabetes, obesity, hypertension, autoimmune disease, sleep disturbance, and chronic inflammation may restrict plasticity by impairing mitochondrial function, perfusion, blood–brain barrier stability, redox balance, or immune resolution. Sex may modulate some of these processes, but systemic health and social determinants can be equally decisive. This is why sex-dependent brain plasticity must be interpreted within a body-brain system.

### 3.5. Interpreting Variability Without Overstatement

**Figure 2:**
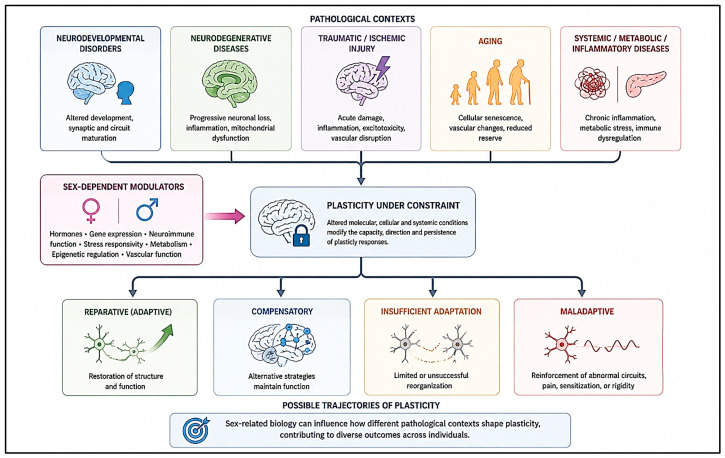
Sex-dependent plasticity under pathological constraint. The diagram is a conceptual synthesis rather than a causal map. Different pathological settings—neurodevelopmental disorders, neurodegenerative diseases, traumatic or ischemic injury, aging, and systemic metabolic or inflammatory disease—place different pressures on plasticity. Sex-dependent biological factors may influence the threshold, direction, energetic cost, persistence, and failure point of plastic responses. Outcomes may be reparative, compensatory, insufficient, or maladaptive, and the same mechanism may shift category over time.

Across these disorders, sex-dependent plasticity is most defensible when it is treated as a modulator of trajectories, not as a binary classifier. It may alter thresholds, timing, pathways, energetic cost, and durability of plastic responses. It may also be absent or clinically negligible in some outcomes. This review therefore explicitly includes neutral and opposing possibilities: sex may matter strongly for one mechanism, weakly for another, and only under specific disease stages or biological states (Figure 2).

## 4. Translational and Therapeutic Implications: From Enhancing Plasticity to Guiding Plasticity

**Figure 3:**
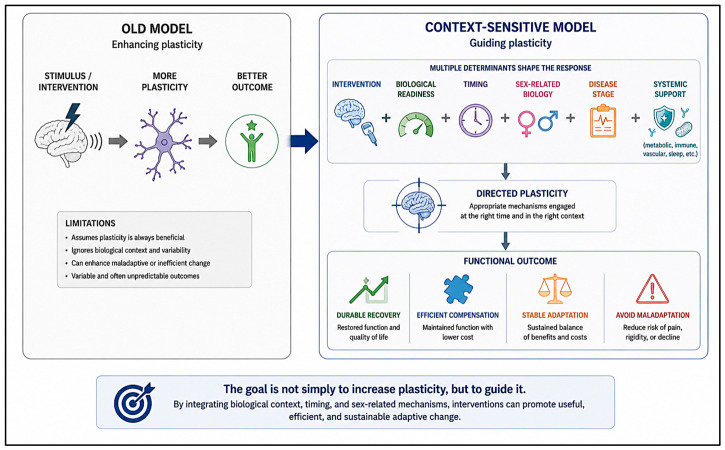
From enhancing plasticity to guiding plasticity. The figure contrasts a simplified model, in which stimulation is assumed to produce more plasticity and therefore better outcome, with a context-sensitive model. In this interpretation, interventions act through biological readiness, timing, sex-related biology, disease stage, systemic support, and monitoring. The figure is conceptual and should not be read as a claim that all listed variables have equal evidential strength or identical effects in all disorders.

To be clinically useful, the idea of guiding plasticity must be made practical. Guiding plasticity means matching intervention type, dose, timing, biological readiness, and monitoring to the state of the nervous system and the body. It also means recognizing that a plasticity-enhancing intervention may be ineffective or harmful if it is applied when fatigue, pain, inflammation, sleep disruption, or metabolic instability prevent consolidation (Table 3 and Figure 3).

Pharmacological treatment offers one example. Sex differences in pharmacokinetics and adverse drug reactions are well documented [45]. For interventions intended to influence excitability, pain, mood, sleep, inflammation, or neurotrophic signaling, equivalent dosing may not produce equivalent biological exposure or equivalent plasticity. This does not justify automatic sex-based dosing, but it supports systematic recording of sex, age, hormonal status, comorbidities, and adverse effects.

Rehabilitation is a second example. Experience-dependent plasticity depends on task specificity, repetition, intensity, salience, and timing [10,46]. However, these principles operate inside a biological organism. A task that is challenging enough to promote learning in one patient may be excessive in another because of pain, poor sleep, fatigue, inflammatory disease, or reduced metabolic reserve. Sex/gender-related differences in fatigue, pain, stress responses, caregiving burden, and rehabilitation access can therefore influence not only outcomes but also the path by which outcomes are achieved.

Post-stroke timing shows why “more and earlier” is not always better. The Critical Period After Stroke Study supports the existence of a sensitive window for additional motor training [47], whereas the AVERT trial showed that very early mobilization at high dose within 24 h was not automatically beneficial and could be harmful depending on intensity and patient state [48]. These findings are not primarily about sex, but they illustrate the broader principle: plasticity must be timed and dosed, not simply maximized. Future trials should ask whether sex-related biology modifies the opening, duration, or consolidation of such windows.

Non-invasive brain stimulation is a third example. rTMS and transcranial direct current stimulation can modulate cortical excitability and have evidence-based indications in several neurological or psychiatric conditions [49,50]. Their effects nevertheless depend on baseline excitability, medication use, sleep, attention, lesion location, network integrity, and timing relative to training. Sex-related differences in endocrine state or cortical excitability may contribute to response variability, but they should be evaluated alongside these other variables.

## 5. Research Priorities and Reporting Standards

Future work should move beyond comparing average male and female outcomes at a single endpoint. A study may find no final sex difference while still missing differences in route, timing, energetic cost, or durability. Conversely, a statistically significant sex difference may be small, context-specific, or partly explained by gender-related exposure, diagnosis, care access, or social behavior. This review therefore recommends trajectory-based designs with repeated measures whenever possible.

Mechanistic studies should specify the level at which sex is expected to matter. If the hypothesis concerns synaptic plasticity, hormonal state and inflammatory context should be considered. If the hypothesis concerns rehabilitation, fatigue, pain, sleep, and metabolic status should not be dismissed as background noise. If the hypothesis concerns neurodegeneration, compensatory plasticity should be examined together with vascular and mitochondrial support.

Reporting standards are essential. In human research, authors should clearly state whether they measured sex, gender, or both; how these variables were assessed; and whether analyses were planned or exploratory [4]. In animal research, ARRIVE 2.0 recommendations support transparent reporting of sex, age, strain, housing, experimental conditions, and design features that affect reproducibility [51]. These standards are especially important in plasticity research because housing, enrichment, stress exposure, lesion model, training intensity, and outcome timing can all change the result.

The figures in this review should also be interpreted with caution. In their legends, empirical and conceptual relationships are differentiated. The figures are intended to help readers organize the argument, not to prove direct causality. Where the evidence is indirect, inconsistent, or disease-specific, the text states this explicitly.

## 6. Conclusions

Brain plasticity is not a uniform therapeutic good. It can repair, compensate, fail, or maladapt. This review makes this distinction operational by defining criteria and examples. It also defines adaptive reserve as a dynamic capacity of neural and systemic biological supports, rather than as a synonym for cognitive reserve or resilience.

Biological sex can influence several mechanisms relevant to plasticity, including endocrine signaling, synaptic remodeling, immune activity, mitochondrial and vascular support, stress regulation, and epigenetic persistence. These influences are real but not deterministic. They are shaped by age, brain region, developmental history, disease stage, systemic health, treatment exposure, and gender-related social context.

The practical implication is that plasticity should be guided rather than indiscriminately enhanced. Guiding plasticity means identifying biological readiness, selecting the right timing and dose, supporting the metabolic and immune conditions needed for useful adaptation, preventing maladaptive stabilization, and monitoring whether improvement is durable or only costly compensation.

A sex-aware view of brain plasticity does not divide neuroscience into rigid male and female categories. It makes plasticity research more biologically honest. It asks why the same lesion, disease burden, intervention, or rehabilitation dose can produce different adaptive trajectories, and it provides a framework for studying those differences without exaggeration.

## Figures and Tables

**Table 1:** Operational criteria used in this review to classify plasticity outcomes.

Plasticity Outcome	Operational Criteria	Examples	Main Caveat
Reparative/adaptive	Improves function with evidence of more efficient or restored neural organization, tissue repair, or durable learning.	Peri-lesional reorganization after stroke that supports motor recovery; developmental learning within a sensitive period.	Short-term improvement is not enough; durability and functional relevance must be shown.
Compensatory	Maintains performance through alternative strategies or circuits, often with greater effort, energetic cost, or vulnerability to later collapse.	Bilateral or alternative network recruitment in aging or early neurodegeneration; camouflaging strategies in autism.	Can be clinically useful and biologically costly at the same time.
Insufficient	Plasticity is initiated but remains too weak, delayed, unstable, or poorly consolidated to preserve function.	Incomplete motor recovery after stroke when fatigue, inflammation, or metabolic disease limits training effects.	Absence of recovery does not prove absence of plasticity; measurement timing matters.
Maladaptive	Plastic change reinforces abnormal excitability, pain, rigidity, inefficient behavior, or progressive decline.	Central sensitization in chronic pain; post-injury spasticity; seizure-prone network reorganization.	Some mechanisms may be adaptive early and maladaptive later.

**Table 2:** Qualitative appraisal of evidence supporting sex-dependent mechanisms relevant to brain plasticity.

Mechanistic Layer	Evidence Supporting Sex-Related Influence	Relative Strength	Limits and Opposing Considerations
Endocrine-synaptic	Sex steroids modulate excitability, dendritic remodeling, synaptic signaling, and neurotrophin pathways.	Strong for mechanism; context-dependent for clinical prediction.	Effects vary by brain region, age, receptor balance, cycle/reproductive stage, and disease state.
Neuroimmune/glial	Microglial and astrocytic responses differ by sex in development, aging, and disease models.	Moderate to strong biologically.	Direction is not uniform; early immune activity may support repair, chronic activity may impair plasticity.
Mitochondrial/metabolic	Plasticity requires energy; sex- and age-related differences in bioenergetics have been shown in some models.	Strong for plasticity support; moderate for sex-specific causality.	Often indirect; systemic disease and age may dominate over sex.
Vascular/glial support	Neurovascular coupling, BBB integrity, and astrocyte function influence adaptation and recovery.	Moderate.	Sex-specific evidence is less consistent and often disease-specific.
Epigenetic/gene regulation	Epigenetic mechanisms can stabilize plasticity-related gene expression and are influenced by sex-linked biology.	Moderate and mechanistically plausible.	Direct evidence linking sex-specific epigenetic states to functional plasticity remains limited.
Stress-axis regulation	HPA-axis reactivity differs by sex and can alter learning, inflammation, sleep, and energetic load.	Moderate to strong.	Acute and chronic stress have different effects; social and gender-related stressors complicate human interpretation.

**Table 3:** Practical examples of “guiding plasticity” in a sex-aware but non-deterministic framework.

Clinical/Research Setting	What Should Be Guided?	Sex/Gender-Related Variables to Record	Practical Implication
Stroke rehabilitation	Timing, intensity, task specificity, fatigue management, and consolidation.	Sex, age, hormonal/reproductive status when relevant, comorbidities, pre-stroke function, access to rehabilitation, social support.	Avoid assuming that more therapy earlier is always better; monitor whether gains are stable or merely compensatory.
Traumatic brain injury	Cognitive load, sleep, fatigue, pain, emotional regulation, and return-to-activity progression.	Sex, injury mechanism, endocrine symptoms, sleep disruption, headache/pain burden, stress exposure.	Use graded progression and monitor delayed worsening, not only early improvement.
Chronic pain	Safety learning, movement exposure, neuromodulation parameters, and avoidance behavior.	Sex/gender, pain phenotype, mood, sleep, stress burden, medication exposure.	Redirect sensitized circuits rather than simply increasing excitability.
Neurodegeneration	Compensation versus exhaustion, cognitive reserve strategies, metabolic/vascular support.	Sex/gender, age, hormonal history, vascular risk, inflammation, sleep, caregiver and social factors.	Track whether network recruitment is efficient compensation or fragile over-recruitment.
Neuromodulation trials	Baseline excitability, stimulation timing, pairing with training, and durability of effects.	Sex, medication use, sleep, menstrual/reproductive status where appropriate, lesion location, fatigue.	Predefine sex-aware analyses without making sex a simplistic treatment selector.

## Data Availability

No new data were created or analyzed in this study. Data sharing is not applicable to this article.
